# Metabolomic Profile and Cytotoxic Activity of *Cissus* *incisa* Leaves Extracts

**DOI:** 10.3390/plants10071389

**Published:** 2021-07-07

**Authors:** Deyani Nocedo-Mena, María Yolanda Ríos, M. Ángeles Ramírez-Cisneros, Leticia González-Maya, Jessica N. Sánchez-Carranza, María del Rayo Camacho-Corona

**Affiliations:** 1 Facultad de Ciencias Químicas, Universidad Autónoma de Nuevo León, Av. Universidad S/N, Ciudad Universitaria, San Nicolás de los Garza 66451, Nuevo León, Mexico; 2 Centro de Investigaciones Químicas-IICBA, Universidad Autónoma del Estado de Morelos, Av. Universidad 1001, Cuernavaca 62209, Morelos, Mexico; myolanda@uaem.mx (M.Y.R.); angelesrc@uaem.mx (M.Á.R.-C.); 3 Facultad de Farmacia, Universidad Autónoma del Estado de Morelos, Av. Universidad 1001, Cuernavaca 62209, Morelos, Mexico; letymaya@uaem.mx (L.G.-M.); jez_chazaq@hotmail.com (J.N.S.-C.)

**Keywords:** *Cissus* *incisa*, Vitaceae, extracts, untargeted metabolomics, cytotoxicity, network pharmacology

## Abstract

*Cissus* *incisa* leaves have been traditionally used in Mexican traditional medicine to treat certain cancerous illness. This study explored the metabolomic profile of this species using untargeted technique. Likewise, it determined the cytotoxic activity and interpreted all data by computational tools. The metabolomic profile was developed through UHPLC-QTOF-MS/MS for dereplication purposes. MetaboAnalyst database was used in metabolic pathway analysis and the network topological analysis. Hexane, chloroform/methanol, and aqueous extracts were evaluated on HepG2, Hep3B, HeLa, PC3, A549, and MCF7 cancer cell lines and IHH immortalized hepatic cells, using Cell Titer proliferation assay kit. Hexane extract was the most active against Hep3B (IC_50_ = 27 ± 3 μg/mL), while CHCl_3_/MeOH extract was the most selective (SI = 2.77) on the same cell line. A Principal Component Analysis (PCA) showed similar profiles between the extracts, while a Venn diagram revealed 80 coincident metabolites between the bioactive extracts. The sesquiterpenoid and triterpenoid biosynthesis pathway was the most significant identified. The Network Pharmacology (NP) approach revealed several targets for presqualene diphosphate, phytol, stearic acid, *δ*-tocopherol, ursolic acid and *γ*-linolenic acid, involved in cellular processes such as apoptosis. This work highlights the integration of untargeted metabolomic profile and cytotoxic activity to explore plant extracts, and the NP approach to interpreting the experimental results.

## 1. Introduction

Cancer is the second leading cause of death globally, responsible for an estimated 9.6 million deaths in 2018. Although important medical and technological advances have been made, conventional therapies directed against cancer have severe side effects and complications such as serious toxicities and development of resistance. In this point, the exploration and discovery of anticancer drugs from medicinal plants is playing an important role [1]. From ancient times, several medicinal plants have been consumed by patients in order to prevent and treat cancer, as an alternative therapy. These plants have been used because of their wealth in anticarcinogenic and chemoprotective potentials. Natural extracts from medicinal plants are a key source of antitumor agents with applicability in anticancer modern therapy [2]. It is known that the synergistic effects of plant extracts of a group of metabolites on a biological activity can play a role together, rather than as a single compound.

Recently, untargeted metabolomics have become a useful tool for the simultaneous analysis of many compounds in vegetal extracts. In contrast to targeted analyses, this technique allows the uncovering of as many groups of metabolites as possible without necessarily identifying or quantifying a particular compound [3]. Mass Spectrometry (MS) in combination with high-performance chromatographic separation is considered the most universal approach for metabolome purposes by its sensitivity, specificity, and demonstrated efficiency in the analysis of plant metabolomes. Moreover, it is known that multivariate statistical techniques are frequently used in these studies, and for exploratory data analysis the PCA can be successfully applied [3,4].

On the other hand, a novel paradigm called NP has gained appreciation as method for omics data integration and multitarget drug development, which combines network biology and polypharmacology approaches. NP attempts to understand metabolites actions and interactions with multiple targets. Currently, this approach is getting attention in cancer research from natural products, since these products aim multiple protein targets and thus, are linked to many types of cancers [5].

Mexico stands out for its broad culture into traditional medicine. Despite the rich experience regarding the use of plants to treat diseases, very few have been studied regarding their phytochemical and pharmacological content. One under-explored species is *Cissus incisa* (Nutt.) Des Moul. Ex S. Watson (syn. *C. trifoliata*), which belongs to Vitaceae family. This plant is native to southern United States and northern of Mexico. It is fast growing and blooms in the summer. Leaves of this plant are used into traditional Mexican medicine to treat skin infections and tumors [6,7].

Because of our interest in giving scientific authentication and explanation of the traditional use of *C. incisa*, the antibacterial potential of some phytocompounds and extracts have been previously determined [8]. Further investigations on CHCl_3_/MeOH extract led to isolation of several compounds such as: ceramides, cerebrosides, *β*-sitosterol, *β*-sitosterol-D-glucopyranoside, *α*-amyrin-3-*O*-*β*-D-glucopyranoside, and 2,3-dihydroxypropyl tetracosanoate [9,10]. Another study reported the chemical and biological profile of the stems of this plant [11]. In spite of the above, and to the best of our knowledge, there are no previous investigations about the cytotoxic activities related to the leaves of this plant.

Accordingly, in this work, the untargeted metabolomic technique was used to explore three extracts from *C. incisa* leaves, by UHPLC-QTOF-MS/MS. Metabolomic fingerprints were obtained by accurate mass measurements, and multivariate analyzes were applied to determine the phytochemical content of the extracts. In addition, the cytotoxic activity of extracts was evaluated on six human cancer cells lines. The integration of the metabolomic study and the cytotoxic activity revealed the cytotoxic metabolites from the bioactive extracts. Finally, a network pharmacology approach was applied to interpret the experimental results.

## 2. Results and Discussion

### 2.1. Metabolomic Profile Analysis of the Extracts

Metabolomic profiling of the extracts from *C. incisa* leaves by UHPLC-QTOF-MS/MS for dereplication purposes, led to the identification of 171, 260, and 114 metabolites in the hexane, CHCl_3_/MeOH and aqueous extracts, respectively (Tables S1–S3 in Supplementary Material). Putative identification of compounds detected were made consulting several databases, such as: MEDLINE_Metabolites, Dictionary of Natural Products, KNApSAcK, PubChem, LIPID MAPS, and Human Metabolome Database (HMDB).

Based on the normalized areas data, three common primary metabolites were detected as the most abundant among the three extracts: two glycerophospholipids and a fatty acyl glycoside (Tables S1–S3). The percentages of abundance of each phytocompound in the hexane, CHCl_3_/MeOH and aqueous extracts were as follows: (0.7491, 0.4864, 1.1213); (0.7480, 0.4856, 1.1222) and (0.7430, 0.4792, 1.0945), respectively. These results are fully comprehensible because glycerophospholipids are the most plentiful phospholipids localized in large amounts in plant cell membranes. In plants, approximately one-third of the organic phosphorus compounds are found in phosphoglycerolipids. In addition, glycerophospholipids participate in cell signaling and as an anchor for proteins in cell membranes [12]. Fatty acyl glucosides, meanwhile, are amphipathic compounds mainly produced by bacteria, yeast, fungi, marine invertebrates, and plants. Recent studies have demonstrated that they play an important role in plant-insect and plant-fungus interactions [13].

Regarding secondary metabolites, *α*-amyrin acetate and *α*-tocopherolquinone were the most abundant compounds in the hexane extract. In the CHCl_3_/MeOH extract, the most abundant secondary metabolites were kazinol A and ursolic acid 3-*O*-*α*-L-arabinopyranoside. Meanwhile in the aqueous, armillane and chabrosterol were found to be the most plentiful compounds (Tables S1–S3).

**Figure 1:**
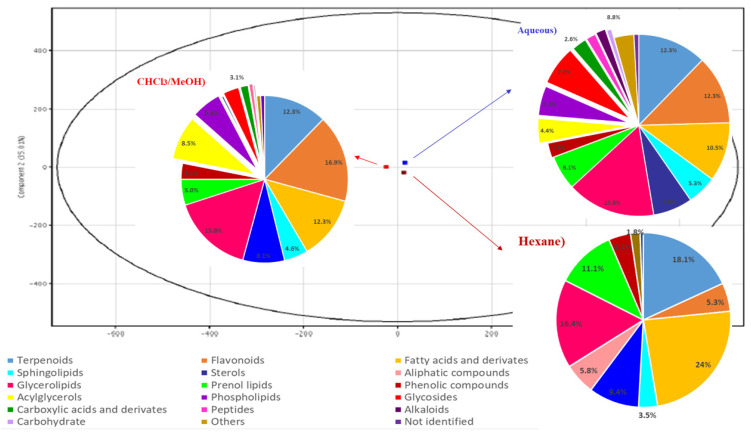
PCA plot of *C. incisa* leaves extracts. PC1 (64.99%), PC2 (35.01%). Within the PCA graph the hexane extract is represented with brown color, CHCl_3_/MeOH in red and the aqueous one in blue. Different classes of metabolites identified in each extract are also represented.

**Figure 2:**
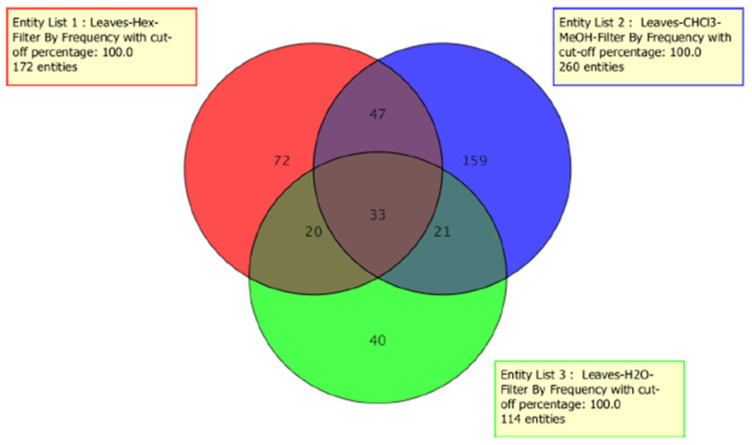
Venn diagram showing the common compounds among the three extracts (*n* = 33), and the common compounds among the most active extracts (hexane and CHCl_3_/MeOH, *n* = 80).

As far as we know, this is the first time that a metabolomic fingerprint of *C. incisa* leaves is reported, thus contributing to the scientific knowledge of this species. A PCA scores plot was obtained (Figure 1) from a multivariate statistical analysis. The PCA showed close metabolomic profiles for the three analyzed extracts. In Figure 1, a similar composition is observed regarding the presence of fatty acyls, sphingolipids, sterols, glycerolipids, prenol lipids, and terpenoids; although their ratio within the extracts is variable. Thirty-three common compounds between these extracts were found (Figure 2, Table S4). Additionally, 80 common compounds were detected only in the hexane and CHCl_3_/MeOH, which are included in Table 1.

The findings presented here agree with those reported by Kumar et al. [14] and Chipiti et al. [15] for the leaf extracts of *C. quadrangularis* and *C. cornifolia*, respectively.

### 2.2. Cytotoxic Activity

Cytotoxic activity of *C. incisa* leaves extracts is also reported here for the first time, which was determined on six human cancer cells. The experimental results are shown in Table 2. According to the National Cancer Institute of the United States of America, an extract is considered active if it achieves an IC_50_ ≤ 30 μg/mL on tumor cells [16]. In this sense, the hexane extract exhibited cytotoxic activity on Hep3B (IC_50_ = 27 ± 3 μg/mL) and HepG2 (IC_50_ = 30 ± 6 μg/mL), being the most active extract. In the case of CHCl_3_/MeOH extract, it was less active on hepatocellular cancer cells, reaching IC_50_ = 39 ± 3 μg/mL and 31 ± 2 μg/mL, respectively. Previously, Opoku et al. [17] reported the antiproliferative activity of MeOH extract of *C. quadrangularis* against the HepG2 cell line with 36.58% of inhibition of proliferation. On the other hand, the hexane extract exhibited certain cytotoxicity on Hela and A549 cancer lines (IC_50_ = 40 ± 2 and 52 ± 2 μg/mL, respectively), similar to the CHCl_3_/MeOH extract against MCF7 (IC_50_ = 50.7 ± 6 μg/mL) and PC3 (57 ± 4 μg/mL).

The Selectivity Index (SI) was determined only for hepatocellular carcinoma cell lines, since they were the most sensitive of all tested (Table 2). It has been reported that SI values less than 2 can indicate toxicity for an extract or a pure compound towards mammal cells [18]. CHCl_3_/MeOH extract gave a SI = 2.77 on Hep3B, and SI = 2.21 on HepG2, surpassing the values of the control (Paclitaxel) on the same cell lines (2.41 and 1.24, respectively). As consequence, the CHCl_3_/MeOH extract from *C. incisa* leaves was the most selective.

The aqueous extract obtained by successive extractions did not show cytotoxic activity in any cancer cell line tested. Different results were obtained by Sáenz et al. [19] evaluating the aqueous extract of *C. sicyoides* leaves (direct extraction) on HEp-2 cells finding a IC_50_ = 43.2 ± 2.4 μg/mL. In addition, our aqueous extract did not show cytotoxicity in immortalized cells (IC_50_ > 100 µg/mL), which is a good first step for further safety studies of the total extract of *C. incisa* aerial parts.

### 2.3. Metabolomics Pathway Analysis

Based on the biological properties displayed by the hexane and CHCl_3_/MeOH extracts, we focused on exploring the 80 common metabolites among these extracts, using the Metabolomics Pathway Analysis (MetPA). As a consequence, the most relevant pathways involving these metabolites were identified, in this case, nine networks were revealed (see in Table 1). The threshold of impact was set to 0.10. The pathway is considered to be closely related if its impact value is higher than this value.

**Figure 3:**
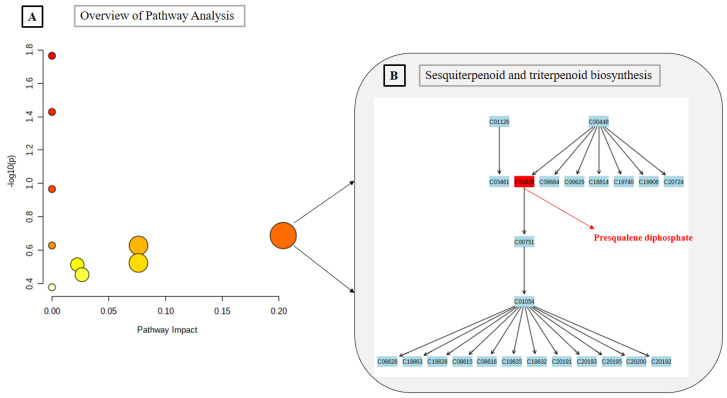
Pathway analysis (**A**) metabolome view, (**B**) pathway with higher impact.

The results obtained from MetPA shows four important routes in plants operation, belonging to their primary metabolism: Linoleic acid metabolism, alpha-Linolenic acid metabolism, Glycerophospholipid metabolism, and Fatty acid biosynthesis. However, the most significant pathway identified via MetPA are those related to the biosynthesis of secondary metabolites, specially terpenes and sterols. This is consistent, since terpenoids and sterols from leaves exhibite a multifunctionality role in plants: more specialized chemical interactions and protection in the abiotic and biotic environment [20]. The results from pathway analysis are presented in detail in Table 3, and only the pathway with higher impact is presented graphically (Figure 3).

### 2.4. Correspondence between Metabolomic Profiling and Cytotoxic Activity

**Figure 4:**
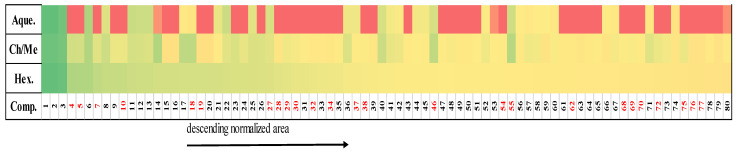
Heat map with the distribution according to the normalized areas of the 80 shared metabolites, identified in the active extracts. Metabolites with previous reports of cytotoxic activity are highlighted in red. Aque *: Aqueous extract; Ch/Me *: CHCl_3_/MeOH extract; Hex *: Hexane extract; Comp *: Compounds: **1**: 1-(1*Z*-octadecenyl)-2-(5*Z*,8*Z*,11*Z*,14*Z*,17*Z*-eicosapentaenoyl)-glycero-3-phospho-(1′-*sn*-glycerol) **2**: 1,2-dihexadecanoylphosphatidylglycerol phosphate; **3**: (3*S*,5*R*,6*S*,7*E*,9x)-7-megastigmene-3,6,9-triol 9-glucoside; **4**: *α*-amyrin acetate; **5**: *α*-tocopherolquinone; **6**: 1-(9*Z*-hexadecenoyl)-2-(11*Z*-eicosenoyl)-glycero-3-phosphoserine; **7**: gibberellin A12 aldehyde; **8**: 16*β*-16-hydroxy-3-oxo-1,12-oleanadien-28-oic acid; **9**: (3*E*)-4-(2,3-dihydroxy-2,5,5,8a-tetramethyl-decahydronaphthalen-1-yl)but-3-en-2-one; **10**: *N*-(3-hydroxy-dodecanoyl)-homoserine lactone; **11**: (all-*E*)-6′-apo-y-caroten-6′-al; **12**: campesteryl *p*-coumarate; **13**: 1-docosanoyl-glycero-3-phospho-(1′-*sn*-glycerol); **14**: 1,2,6a,6b,9,9,12a-heptamethyl-10-[(3,4,5-trihydroxyoxan-2-yl)oxy]-1,2,3,4,4a,5,6,6a,6b,7,8,8a,9,10,11,12,12a,12b,13,14b-icosahydropicene-4a-carboxylate; **15**: 7,9,13,17-tetramethyl-7*S*,14*S*-dihydroxy-2*E*,4*E*,8*E*,10*E*,12*E*,16-octadecahexaenoic acid; **16**: calycanthidine; **17**: not identified; **18**: 5,7,4′-trimethoxyflavan; **19**: alpinumisoflavone dimethyl ether; **20**: gancaonin R; **21**: *β*-citraurinene; **22**: spheroidenone; **23**: 6-deoxohomodolichosterone; **24**: 3*β*,18*β*-3-methoxy-11-oxo-12-oleanen-30-oic acid; **25**: (5*α*,25*R*)-spirostan-3,6-dione; **26**: 5,6-epoxy-5,6-dihydro-10′-apo-*β*,γ-carotene-3,10′-diol; **27**: stylisterol B; **28**: stylisterol A; **29**: grandifloric acid; **30**: phytol; **31**: 7′,8′-Dihydro-8′-hydroxycitraniaxanthin; **32**:*γ*-linolenic acid; **33**: Amabiline; **34**: **c**ucurbitacin E; **35**: 2-stearyl citric acid; **36**: (1-cyano-2-methylprop-2-en-1-yl) 9Z,12Z-octadecadienoate; **37**: ursolic acid; **38**: *ent*-9-L1-phytoP; **39**: 17-phenyl heptadecanoic acid; **40**: 1-dodecanoyl-glycero-3-phospho-(1′-*sn*-glycerol); **41**: 14-*O*-(*α*-L-rhamnopyranosyl)-7*S*,14*R*-dihydroxy-7,9,13,17-tetramethyl-2*E*,4*E*,8*E*,10*E*,12*E*,16*E*-octadecahexaenoic acid; **42**: 1-octadecanoyl-2-docosanoyl-*sn*-glycero-3-phosphate; **43**: 10-methoxyheptadec-1-en-4,6-diyne-3,9-diol; **44**: (12*S*,15*S*)-15-*O*-demethyl-10,29-dideoxy-11,12-dihydro-striatin C; **45**: fragarin; **46**: 5-methoxy-3-(2*R*-acetoxy-pentadecyl)-1,4-benzoquinone; **47**: Yucalexin B16; **48**: Junceic acid; **49**: Yucalexin B5; **50**: *N*-tetradecanoyl glutamine; **51**: 10,13-Epoxy-11-methyloctadeca-10,12-dienoic acid; **52**: 19*α*-19-hydroxy-3,11-dioxo-12-ursen-28-oic acid; **53**: 1-monoacylglycerol; **54**: (−)-folicanthine; **55**: 7-oxo-*β*-sitosterol; **56**: 1-dodecanoyl-*sn*-glycero-3-phosphocholine; **57**: 1-(9*Z*,12*Z*-octadecadienoyl)-*rac*-glycerol; **58**: 1-(11*Z*,14*Z*-eicosadienoyl)-glycero-3-phosphate; **59**: Flavoxate; **60**: (−)-Epicatechin 3′-*O*-sulfate; **61**: 2-Heptadecylfuran; **62**: matricin; **63**: heneicosan-2-one; **64**: austroinulin; **65**: heliotrine; **66**: doristerol; **67**: crispane; **68**: stearic acid; **69**: 2-hydroxy-6-tridecylbenzoic acid; **70**: stigmastane-3,6-dione; **71**: 1-pentadecanoyl-2-arachidonoyl-*sn*-glycero-3-phosphate; **72**: oxyacanthine; **73**: all-*trans*-Heptaprenyl diphosphate; **74**: cavipetin D; **75**: presqualene diphosphate; **76**: lucidone A; **77**: *δ*-tocopherol; **78**: 1-(4*Z*,7*Z*,10*Z*,13*Z*,16*Z*,19*Z*-docosahexaenoyl)-2-(13*Z*-docosenoyl)-*sn*-glycero-3-phosphocholine; **79**: (22*E*,24*R*)-stigmasta-4,22-diene-3,6-dione; **80**: 2-monopalmitoylglycerol. * tentative assignment based on accurate mass.

The distribution of the 80 coincident compounds is presented in a heat map (Figure 4), which contains the normalized relative areas of these metabolites, identified in the hexane, CHCl_3_/MeOH and the aqueous extracts. The heat map also shows the distribution between the three extracts of the cytotoxic metabolites reported against the same cell lines included in this study (or some related ones). It can be seen that most of the cytotoxic compounds are found in a higher proportion within the hexane extract.

As it presented in Table 2, hexane and CHCl_3_/MeOH extracts had similar cytotoxic results on the hepatocellular cells (even if the hexane extract was more active on Hep3B). These similarities can be explaining by the chemical content, these extracts include 80 common metabolites (Venn diagram Figure 2), showing a correspondence between the metabolomics profiles of the active extracts and the cytotoxic activity on Hep3B and HepG2 cell lines. These cells share common characteristics (for instance Wnt/β-catenin activation [21], providing a unique platform for parallel comparisons, but also HepG2 and Hep3B are from different ethnic origins. Some differential gene expression (for instance; HepG2 cells are known to contain wild-type p53 whereas Hep3B cells are p53 deficient), provide a broad spectrum of mechanisms, particularly for apoptosis induction. Several studies suggested that phytosterols and terpenes disturb the cell cycle and induce apoptosis by activating caspases 3 and 9 in cancer cells. Particularly triterpenes and its derivates glycosides have shown effect against cancer cells and induction of apoptosis mechanism [22]. These phytocompounds are present in both extracts (hexane and CHCl_3_/MeOH) (Figure 4).

It is necessary to point out that some of these 80 shared metabolites have been previously reported with cytotoxic activity against hepatocellular cancer cells: (**5**) *α*-tocopherolquinone, (**30**) phytol, (**29**) grandifloric acid, (**34**) cucurbitacin E, (**4**) *α*-amyrin acetate, (**37**) ursolic acid, (**32**) *δ*-linolenic acid, (**72**) oxyacanthine, (**68**) stearic acid, and (**62**) matricin of which, the first six are terpenoids, including three triterpenes. The presence of these cytotoxic metabolites may explain the cytotoxicity of the extracts (numbering is according to the heat map, Figure 4).

*α*-Tocopherolquinone was dereplicated with molecular formula (C_29_H_50_O_3_) and accurate mass 446.3760. This diterpene has reported good cytotoxic activity on HepG2 cells (IC_50_ = 6.97 ± 0.5 µg/mL) [23]. Another terpene dereplicated, phytol (C_20_H_40_O; accurate mass 296.3079) selectively inhibited the growth of the HepG2 cells with an IC_50_ value of 78 ± 3.45 μM [24]. Another study showed that phytol exerted antitumor effect in hepatocellular carcinoma cells by activation of caspases 9/3 [25]. The triterpene cucurbitacin E (formula suggested C_32_H_44_O_8_; accurate mass 556.3036) exhibited antiproliferative action on Hep3B cancer cells through inhibition of Wnt/β-catenin activation [26]. Meanwhile, C_32_H_52_O_2_ (468.3967) identified as *α*-amyrin acetate, and showed moderate activity on HepG2 = 148.9 ± 1.80 µM [27]. Other triterpene, ursolic acid (C_30_H_48_O_3_; accurate mass 456.3603) is distributed among the three extracts; it has been widely studied in relation with anticancer properties. In Hep3B cell lines, ursolic acid has reduced the tumorigenesis in vivo, enhancing apoptosis in tumor tissues [28], and exerting antiangiogenic action [29]. A different work showed that ursolic acid displayed effects on cell viability, DNA fragmentation, mitochondrial membrane potential on human liver cancer HepG2 (IC_50_ = 4 μM) and Hep3B (IC_50_ = 8 μM) cells [30]. A study showed in vivo that *γ*-linolenic acid (C_18_H_30_O_2_; 278.2246) reduced the proliferative and angiogenic effect of carcinoma hepatocellular induced in Wistar rats, by activation of a mitochondrial mediated apoptosis pathway [31]. Likewise, oxyacanthine (C_37_H_40_N_2_O_6_, 608.28863) attenuated cell proliferation ability and promoted cell apoptosis in mammary, prostatic, liver cancers cells [32], while stearic acid [33] (C_18_H_36_O_2_; 284.2715) and grandifloric acid [34] (terpene; C_20_H_30_O_3_; 318.2194) had the same action on HepG2 cells. A recent study determined the antiproliferative activity of extracts from Australian plants leaves that contained matricin (C_17_H_22_O_5_; 306.1467), a prenol lipid, on HepG2 cells [35].

Other dereplicated compounds with promising anticancer activities reported are: (**19**) alpinumisoflavone dimethyl ether (C_22_H_20_O_5_; 364.1311) that suppress the proliferation, migration/invasion, tumor angiogenesis and metastasis, and the promotion of apoptosis in various cancers: human oral epidermoid carcinoma KB cells (IC_50_ = 4.13 μg/mL), and murine leukemia P-388 (IC_50_ = 4.31 μg/mL) cells [36] and (**10**) *N*-(3-hydroxy-dodecanoyl)-homoserine lactone (C_16_H_29_NO_4_; 299.2097) with pro-apoptotic activities [37] (**28**) Stylisterol A (C_28_H_46_O_3_; 430.3447), (**27**) stylisterol B (C_28_H_46_O_4_; 446.3396), [38] and (**7**) gibberellin A12 aldehyde [39] (C_20_H_28_O_3_; 316.2038) have been found to have antiproliferative action in cancer cells, even with apoptosis induction.

In the heat map (Figure 4), it can be observed that the aforementioned metabolites occur in the hexane extract, justifying why this extract is the most active of the three tested. While, only (**55**) 7-oxo-*β*-sitosterol [40] (C_29_H_48_O_2_; 428.3654), (**46**) 5-methoxy-3-(2*R*-acetoxy-pentadecyl)-1,4-benzoquinone [41] (C_24_H_38_O_5_; 406.2719), (**69**) 2-hydroxy-6-tridecylbenzoic acid [42] (C_20_H_32_O_3_; 320.2351), (**70**) stigmastane-3,6-dione [43] (C_29_H_48_O_2_; 428.3654), and (**18**) 5,7,4′-trimethoxyflavan [44] (C_18_H_20_O_4_; 300.1362) appear preferably in the CHCl_3_/MeOH extract, and have also displayed anticancer effects. Therefore, all the metabolites presented so far are involved in the cytotoxic activity of the active extracts.

On the other hand, there are other experimental results from cytotoxic assays that are worth discussing. As we presented earlier, two cell lines (HeLa and A549) were more susceptible to hexane extract than CHCl_3_/MeOH extract (Table 2). In this regard, the fold change analysis detected 30 up-regulated phytocompounds in hexane extract (Table 4). In contrast, PC3 and MCF7 cell lines were more sensitive to the CHCl_3_/MeOH extract than the hexane one according to Table 2. Thirty-eight up-regulated compounds were identified in the CHCl_3_/MeOH extract by the fold change analysis. Table 4 also contains these compounds, along with the previous studies against Hela, A549, MCF7 and/or related cell lines.

### 2.5. Network Pharmacology (NP)

**Figure 5:**
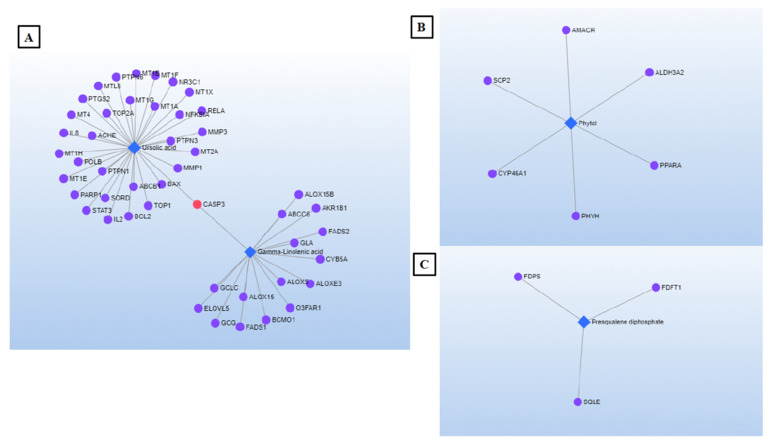
(**A**–**C**). Graphs of the selected interaction networks.

NP approach was used to explore metabolite/gen/disease interaction in the cancer context. The results displayed the synergist activity of some metabolites to achieve anticancer effect. Some compounds such as presqualene diphosphate, phytol, stearic acid, *δ*-tocopherol, ursolic acid and *γ*-linolenic acid are directly involved in the five sub-networks identified. Figure 5A–C shows three interaction networks selected. Figure 5A is about the most noteworthy network by the largest number of concerned nodes. Some key genes identified in this network are recognized for the National Center for Biotechnology Information (NCBI) for their significant role in drug discovery [53]: CASP3 (caspase 3), the protein encoded by this gene plays a central role in the execution-phase of cell apoptosis. In addition, two nuclear receptors (PARP1; NR3C1) involved in the regulation of several important cellular processes such as differentiation, proliferation, and in the recovery of cell from DNA damage. Likewise, three different genes (BAX, BCL2, STAT3) whose encoded proteins were implicated in cell growth and apoptosis. Two well-known signaling molecules (PTPN6 and PTPN3) were also identified, which regulate a variety of cellular processes including cell growth, differentiation, mitotic cycle, and oncogenic transformation. Last, DNA topoisomerase (TOP2A) was also recognized. The gene encoding this enzyme functions as the target for several anticancer agents and a variety of mutations in this gene have been associated with the development of drug resistance [53].

The analysis of the pharmacological network revealed, as targets, several genes involved in the inflammatory response, which occurs in various pathological conditions, such as cancer (CXCL8, ALOX5 and ALOX15) [53]. Phytol (network Figure 5B), targets PPARα. This gene is implied in cell proliferation, cell differentiation, and immune and inflammation responses. Along with presqualene diphosphate (Figure 5C), phytol targets genes encoding proteins involved in drug metabolism and synthesis of cholesterol, steroids, and other lipids (CYP46A1 and FDFT1, respectively) [53].

Summarizing, the current work presents for the first time the metabolomic fingerprint of *C. incisa* leaves, and the cytotoxic properties of their extracts. Untargeted metabolomics profiles through UHPLC-QTOF-MS and multivariate analyzes allowed to determine the phytochemical similarities and differences between the three extracts and to understand their cytotoxic effects by the presence of bioactive metabolites. The hexane extract achieved remarkable cytotoxicity on hepatocellular cancer cells, hence, coupling the metabolome data with its biological activity could support a targeted isolation focused on the predicted active metabolites. The NP approach used was successful for the interpretation of the experimental results, because the metabolites that contribute to the cytotoxic activity and the molecular pathways involved were revealed.

## 3. Materials and Methods

### 3.1. Vegetal Material and Extracts Preparation

*Cissus incisa* (Nutt.) Des Moul. Ex S. Watson was collected in Rayones, Nuevo Leon, Mexico (Latitude: 25.0167°, Longitude: −100.05°, Altitude: 900 m) on 10 October 2016. The identification was made by the biologist Ph.D. Mauricio Gonzalez Ferrara (Autonomous University of Nuevo Leon, San Nicolás de los Garza, Mexico). The collected species were deposited in the herbarium of Biological Sciences Faculty of the Autonomous University of Nuevo Leon with Voucher 027499. The plant name has been checked with http://www.theplantlist.org, accessed on 3 February 2020.

Leaves were dried at room temperature for 2 weeks and then milled until obtaining 809 g of dried and grounded plant material. Sequential macerations were made using hexane (10 L), chloroform/methanol (1:1) (7 L), and water (10 L) yielding the organic extracts and the aqueous extract. The extractions were made at room temperature, following the same steps: filtration and vacuum distillation to dryness for the organic extracts. Whereas, a lyophilization was carried out to obtain the dry aqueous extract, yielding 11.6 g of hexane (1.43%), 84 g of CHCl_3_/MeOH (10.38%), and 19.6 g of aqueous one (2.42%) of dry extracts.

### 3.2. UHPLC-QTOF-MS/MS Analysis

All solvents LCMS grade Baker (Thermo Fisher Scientific, Waltham, MA, USA) were filtered using membrane filter, NYLON 0.45-micron × 47 mm (DS0215-4045, Thermo Fisher Scientific, Waltham, MA, USA). Three samples per extract were diluted independently (1 mg/mL) in MeOH (50%), sonicated 5 min × 10,000 rpm and filtered using PTFE 0.20 µm Syringe filter (721-1320 Thermo Scientific, Waltham, MA, USA), and transferred to a high-recovery MS Analyzed Type 1 borosilicate amber glass vial (5190-7041/5182-0717, Agilent Technologies, Santa Clara, CA, USA).

Reverse-phase liquid chromatography was performed at 20 °C, using an Agilent 1290 Infinity II Ultra High-Performance Liquid chromatography system (UHPLC Waters, Singapore, Singapore) and the column ZORBAX Eclipse Plus C18 HD 2.1 × 50 mm, 1.8 µm (Agilent Technologies, Santa Clara, CA, USA). The mobile phase was delivered by a binary pump at a flow rate of 0.250 mL/min in a gradient elution using: LCMS grade water + 0.1% *v*/*v* formic acid (solvent A) and LCMS grade MeOH + 0.1% *v*/*v* formic acid (solvent B) with the following gradient conditions: 0–6 min, from 30 to 100% solvent B; held at 100% B until 10 min; 10–11 min, from 100 to 30% B to return to original conditions. Injection volume was 5 µL. Mass spectrometric analysis was performed using an Agilent 6545 Quadrupole Time of Flight (QTOF) LCMS with an electrospray ionization (ESI) source (Agilent Technologies, Waldbronn, Germany), in positive mode. Detection range of mass-to-charge ratio (*m*/*z*) was 100–3000. The nebulizer pressure was set at 35 psi, gas temperature of 320 °C, and a gas flow rate of 8 L/min.

### 3.3. Data Processing and Metabolic Pathway Analysis

The identification of metabolites was carried out using the METLIN_Metabolites Database on Agilent MassHunter Qualitative Analysis B.08.00 software and the lists for data analysis were generated with compounds present in all the replicates of each extract. Putative assignments for each compound were made based on their accurate mass. Additionally the Dictionary of Natural Products, PubChem, (http://pubchem.ncbi.nlm.nih.gov/, accessed on 11 October 2020), LIPID MAPS (http://www.lipidmaps.org/tools, accessed on 11 October 2020), and Human Metabolome Database (HMDB) (http://www.hmdb.ca, accessed on 11 October 2020) were consulted. Principal component analysis (PCA), Venn diagram, and fold change analysis (cut off 2.0) were carried out for UHPLC-QTOF-MS/MS data on Mass Profiler Professional software. PCA presents the average of replicates by each extract. For all statistical tests performed, ANOVA with cut-off *p* < 0.05 was taken as significant. The metabolomics pathway analysis and the network topological analysis were performed with MetaboAnalyst (http://www.metaboanalyst.ca/, accessed on 3 February 2021) and STITCH (http://stitch.embl.de/, accessed on 3 February 2021). The metabolite-gene-disease interaction network was selected within the MetPA module, through the integration of network topological analysis, interactive network exploration, and functional enrichment analysis.

### 3.4. Cytotoxic Activity

#### 3.4.1. Cell Lines

The extracts were evaluated for their cytotoxic activity in human cancer cells: PC3 (prostate ATCC^®^ CRL-1435), Hep3B (hepatocellular ATCC^®^ HB-8064), and HepG2 (hepatocellular ATCC^®^ HB-8065), MCF7 (breast (ATCC^®^ HTB-22), A549 (lung (ATCC^®^ CCL-185), and HeLa (cervical ATCC^®^ CCL-2), all were obtained from ATCC (American Type Culture Collection, Manassas, VA, USA). In addition, a cell line of immortalized human hepatocytes (IHH) was included as control of non-cancerous cells [54]. PC3 cells were cultured in RPMI-1640 medium (Sigma Aldrich, St. Louis, MO, USA), while Hep3B, HepG2, IHH, MCF7, A549 and HeLa in DMEM (Invitrogen, Thermo Fisher Scientific, Inc., Waltham, MA, USA) and supplemented with 10% fetal bovine serum (SFB, Invitrogen, Waltham, MA, USA) and with 2 mM glutamine, all cultures were incubated at 37 °C in a 5% CO_2_ atmosphere.

#### 3.4.2. IC_50_ Determination

For the cytotoxic evaluation 4000 cells were cultured per well in 96-well plates. The concentrations used for the extracts and for positive control Paclitaxel were 100, 10, 1, 0.1, 0.01 μg/mL for a dose/response curve.

Prior to the assay, stock solutions of 20 mg/mL (20,000 µg/mL) of each extract were prepared, (1 mg of extract dissolved in 50 µL of DMSO) for organic extracts and sterile water for the aqueous one.

The solutions were prepared from this stock as follows. The concentration 100 µg/mL was prepared from 2.5 µL of a stock solution 20 mg/mL (20,000 µg/mL) in 497.5 µL of culture medium. The concentration 10 µg /mL was prepared from 50 µL of the 100 µg/mL solution in 450 µL of medium. The concentration 1 µg/mL was prepared from 50 µL of the 10 µg/mL solution in 450 µL of medium. The concentration 0.1 µg/mL was prepared from 50 µL of the 1 µg/mL solution in 450 µL of medium. The concentration 0.01 µg/mL was prepared from 50 µL of the 0.1 µg/mL solution in 450 µL of medium.

Subsequently, 100 µL of each solution was added to its corresponding well. Treatment with extracts did not exceed 0.5% of DMSO. In addition, a solvent control was performed at this concentration, not observing cell growth inhibition, which guaranteed that the cytotoxic activity of each extract was associated with the chemical content present in each extract and not with the solvent.

Plates were incubated at 37 °C in 5% CO_2_ atmosphere for 48 h. The number of viable cells in proliferation was then determined by the Cell Titer 96^®^ aqueous solution cell proliferation assay kit (Promega, Madison, WI, USA) following the supplier’s protocol. Cell viability was determined by absorbance at 450 nm using an automated ELISA reader. The experiments were performed in triplicate in three independent experiments. Data were expressed as means ± SD and were analyzed in the Prism 5.0 statistical program, IC_50_ values were determined by regression analysis [54].

#### 3.4.3. Selectivity Index

The extracts were tested against IHH normal cell line [54] to determine the selectivity of the cytotoxic activity on hepatocellular lines. The Selectivity Index (SI) was calculated following previous reports [55]: SI = IC_50_ of extract in a normal cell line/IC_50_ of the same extract in cancer cell line, where IC_50_ is the concentration required to kill 50% of the cell population.

## Figures and Tables

**Table 1:** Common compounds identified in the hexane and CHCl_3_/MeOH extracts (UHPLC-QTOF-MS/MS)- and metabolomic pathways.

Identified Metabolites	Molecular Formula	Accurate Mass	Metabolite Class	Related Pathway
*α*-Tocopherolquinone *	C_29_H_50_O_3_	446.3760	Diterpenoid	
Alpinumisoflavone dimethyl ether *	C_22_H_20_O_5_	364.1311	Flavonoid	
7,9,13,17-tetramethyl-7*S*,14*S*-dihydroxy-2*E*,4*E*,8*E*,10*E*,12*E*,16-octadecahexaenoic acid *	C_22_H_32_O_4_	360.2301	Fatty acid derivative	
7-oxo-*β*-Sitosterol	C_29_H_48_O_2_	428.3654	Sterol	
Heneicosan-2-one *	C_21_H_42_O	310.3236	Fatty Acyl	
Gancaonin R *	C_24_H_30_O_4_	382.2144	Stilbene	
1-Monoacylglycerol	C_21_H_36_O_4_	352.2614	Acyl glycerol	
14-*O*-(*α*-L-rhamnopyranosyl)-7*S*,14*R*-dihydroxy-7,9,13,17-tetramethyl-2*E*,4*E*,8*E*,10*E*,12*E*,16*E*-octadecahexaenoic acid	C_28_H_42_O_8_	506.2880	Fatty acid glycoside	
Calycanthidine *	C_23_H_28_N_4_	360.2314	Alkaloid	
1,2,6*α*,6*β*,9,9,12*α*-Heptamethyl-10-[(3,4,5-trihydroxyoxan-2-yl)oxy]-1,2,3,4,4*α*,5,6,6*α*,6*β*,7,8,8*α*,9,10,11,12,12*α*,12*β*,13,14*β*-icosahydropicene-4*α*-carboxylate	C_35_H_56_O_7_	588.4026	Terpenoid	
2-Heptadecylfuran *	C_21_H_38_O	306.2923	Heteroaromatic compound	
5-Methoxy-3-(2*R*-acetoxy-pentadecyl)-1,4-benzoquinone	C_24_H_38_O_5_	406.2719	Quinone	
Phytol *	C_20_H_40_O	296.3079	Diterpenoid	
Oxyacanthine *	C_37_H_40_N_2_O_6_	608.2886	Lignan	
Yucalexin B16 *	C_20_H_28_O_2_	300.2089	Diterpenoid	
Campesteryl *p*-coumarate	C_37_H_54_O_3_	546.4072	Steroid ester	
1-dodecanoyl-glycero-3-phospho-(1′-*sn*-glycerol)	C_18_H_37_O_9_P	428.2175	Glycerophospholipid	
10,13-Epoxy-11-methyloctadeca-10,12-dienoic acid *	C_19_H_32_O_3_	308.2351	Fatty Acyl derivative	
Spheroidenone	C_41_H_58_O_2_	582.4437	Carotene derivative	
2-Monopalmitoylglycerol	C_19_H_38_O_4_	330.2771	Monoglyceride	
Doristerol	C_27_H_46_O	386.3549	Sterol	
(12*S*,15*S*)-15-*O*-demethyl-10,29-dideoxy-11,12-dihydro-striatin C	C_25_H_38_O_6_	434.2668	Terpene	
*δ*-Tocopherol *	C_27_H_46_O_2_	402.3498	Prenol lipid	Ubiquinone and other terpenoid-quinone biosynthesis
16*β*-16-Hydroxy-3-oxo-1,12-oleanadien-28-oic acid	C_30_H_44_O_4_	468.3240	Triterpene	
(3*E*)-4-(2,3-dihydroxy-2,5,5,8*α*-tetramethyl-decahydronaphthalen-1-yl)but-3-en-2-one *	C_18_H_30_O_3_	294.2195	Sesquiterpenoid	
3*β*,18*β*-3-Methoxy-11-oxo-12-oleanen-30-oic acid *	C_31_H_48_O_5_	484.7104	Triterpenoid	
(1-cyano-2-methylprop-2-en-1-yl) 9Z,12Z-octadecadienoate	C_23_H_37_NO_2_	359.2824	Fatty Acyl	
Fragarin	C_21_H_21_O_10_	434.1207	Flavonoid	
Flavoxate	C_24_H_25_NO_4_	391.1784	Flavonoid	
*β*-Citraurinene	C_30_H_42_O	418.3236	Triterpenoid	
*N*-(3-hydroxy-dodecanoyl)-homoserine lactone *	C_16_H_29_NO_4_	299.2097	Fatty Acyl	
1-(11*Z*,14*Z*-eicosadienoyl)-glycero-3-phosphate	C_23_H_43_O_7_P	462.2746	Glycerophospholipid	Glycerophospholipid metabolism
Cavipetin D	C_25_H_38_O_5_	418.2719	Diterpenoid	
10-Methoxyheptadec-1-en-4,6-diyne-3,9-diol *	C_18_H_28_O_3_	292.2038	Fatty Acyl	
1-pentadecanoyl-2-arachidonoyl-*sn*-glycero-3-phosphate	C_38_H_67_O_8_P	682.4574	Glycerophospholipid	
Diisobutyl phthalate	C_16_H_22_O_4_	278.1516	Pollutant	
Lucidone A *	C_24_H_34_O_5_	402.2406	Sesquiterpenoid	
1-(9*Z*,12*Z*-octadecadienoyl)-*rac*-glycerol	C_21_H_38_O_4_	354.2770	Glycerolipid	
(3*S*,5*R*,6*S*,7*E*,9x)-7-Megastigmene-3,6,9-triol9-glucoside	C_19_H_34_O_8_	390.2253	Fatty acyl glycosides	
5,7,4′-Trimethoxyflavan	C_18_H_20_O_4_	300.1362	Flavonoid	
*all*-*trans*-Heptaprenyl diphosphate *	C_35_H_60_O_7_P_2_	654.3814	Prenol lipid	
1-(1*Z*-octadecenyl)-2-(5*Z*,8*Z*,11*Z*,14*Z*,17*Z*-eicosapentaenoyl)-glycero-3-phospho-(1′-*sn*-glycerol)	C_44_H_77_O_9_P	780.5305	Glycerophospholipid	
Heliotrine *	C_16_H_27_NO_5_	313.1889	Member of pyrrolizines	
(*all*-*E*)-6′-Apo-y-caroten-6′-al	C_32_H_42_O	442.3272	Prenol lipid	
1-dodecanoyl-*sn*-glycero-3-phosphocholine	C_20_H_42_NO_7_P	439.2699	Glycerophospholipid	
1-(9*Z*-hexadecenoyl)-2-(11*Z*-eicosenoyl)-glycero-3-phosphoserine	C_42_H_78_NO_10_P	787.5363	Glycerophospholipid	
Stylisterol B	C_28_H_46_O_4_	446.3396	Sterol Lipid	
Stylisterol A *	C_28_H_46_O_3_	430.3447	Sterol Lipid	
Cucurbitacin E *	C_32_H_44_O_8_	556.3036	Triterpenoid	
(22*E*,24*R*)-Stigmasta-4,22-diene-3,6-dione	C_29_H_44_O_2_	424.3341	Lipid	
Junceic acid *	C_21_H_30_O_3_	330.2195	Prenol lipid	
2-Hydroxy-6-tridecylbenzoic acid *	C_20_H_32_O_3_	320.2351	phenolic compound	
1-docosanoyl-glycero-3-phospho-(1′-*sn*-glycerol)	C_28_H_57_O_9_P	568.3740	Glycerophospholipid	
Gibberellin A12 aldehyde *	C_20_H_28_O_3_	316.2038	Prenol lipid	Diterpenoid biosynthesis
Matricin *	C_17_H_22_O_5_	306.1467	Prenol lipid	
19-*α*-19-hydroxy-3,11-dioxo-12-ursen-28-oic acid	C_30_H_44_O_5_	484.3188	Triterpenoid	
*α*-Amyrin acetate *	C_32_H_52_O_2_	468.3967	Triterpenoid	
(5*α*,25*R*)-Spirostan-3,6-dione	C_27_H_40_O_4_	428.2926	Sterol	
*Ent*-9-L1-phytoP *	C_18_H_28_O_4_	308.1988	Fatty Acyl	
*γ*-Linolenic Acid *	C_18_H_30_O_2_	278.2246	Fatty acid	Biosynthesis of unsaturated fatty acids
Crispane	C_20_H_32_O_3_	320.2351	Terpene	
Austroinulin *	C_20_H_34_O_3_	322.2508	Diterpenoid	
Presqualene diphosphate *	C_30_H_52_O_7_P_2_	586.3188	Terpenoid	Sesquiterpenoid and triterpenoid biosynthesis; Steroid biosynthesis
(−)-Folicanthine *	C_24_H_30_N_4_	374.2470	Indoles derivative	
1-Octadecanoyl-2-docosanoyl-*sn*-glycero-3-phosphate	C_43_H_85_O_8_P	760.5982	Glycerophospholipid	
Ursolic acid	C_30_H_48_O_3_	456.3603	Triterpenoid	
Amabiline *	C_15_H_25_NO_4_	283.1784	Carboxylic ester	
(−)-Epicatechin 3′-*O*-sulfate	C_15_H_14_O_9_S	370.0358	Flavonoid	
Stearic acid *	C_18_H_36_O_2_	284.2715	Fatty acid	Biosynthesis of unsaturated fatty acids
Grandifloric acid *	C_20_H_30_O_3_	318.2194	Terpene	
1,2-Dihexadecanoylphosphatidylglycerol phosphate	C_38_H_76_O_13_P_2_	802.4761	Glycerophospholipid	
Yucalexin B5 *	C_20_H_26_O_3_	314.1881	Terpene	
6-Deoxohomodolichosterone *	C_29_H_50_O_4_	462.3709	Sterol Lipid	
7′,8′-Dihydro-8′-hydroxycitraniaxanthin *	C_33_H_44_O_3_	488.3290	Triterpenoid	
5,6-Epoxy-5,6-dihydro-10′-apo-*β*,γ-carotene-3,10′-diol *	C_27_H_38_O_3_	410.2820	Carotenoid	
*N*-tetradecanoyl glutamine *	C_19_H_36_N_2_O_4_	356.2675	Fatty Acyl	
Stigmastane-3,6-dione *	C_29_H_48_O_2_	428.3654	Sterol Lipid	
2-Stearyl citric acid *	C_24_H_44_O_7_	444.3087	Tricarboxylic acid	
1-(4*Z*,7*Z*,10*Z*,13*Z*,16*Z*,19*Z*-Docosahexaenoyl)-2-(13*Z*-docosenoyl)-*sn*-glycero-3-phosphocholine *	C_52_H_90_NO_8_P	887.6404	Glycerophospholipid	
17-Phenyl heptadecanoic acid *	C_23_H_38_O_2_	346.2872	Fatty Acyl	

* Not detected in aqueous extract.

**Table 2:** Cytotoxic activity of *C. incisa* leaves extracts.

Cell Lines	Hexane Extract		CHCl_3_/MeOH Extract		Aqueous Extract		Paclitaxel	
	IC_50_ (µg/mL)	SI	IC_50_ (µg/mL)	SI	IC_50_ (µg/mL)	SI	IC_50_ (µg/mL)	SI
HepG2	30 ± 6	1.5	39 ± 3	2.21	>100	ND	64 × 10^−3^	1.24
Hep3B	27 ± 3	1.66	31 ± 2	2.77	>100	ND	33 × 10^−3^	2.41
HeLa	40 ± 2	ND	61 ± 4	ND	>100	ND	4.78 × 10^−3^	ND
A549	52 ± 2	ND	77 ± 6	ND	>100	ND	5.12 × 10^−3^	ND
PC3	76 ± 5	ND	57 ± 4	ND	>100	ND	10.2 × 10^−3^	ND
MCF7	74 ± 6	ND	50.7 ± 6	ND	>100	ND	4.27 × 10^−3^	ND
IHH	45 ± 3		86 ± 5		>100		79.4 × 10^−3^	

Values expressed are ±SD of three independent experiments (*n* = 3); ND = not determined.

**Table 3:** Results from Pathway Analysis with MetaboAnalyst.

No.	Pathway Name	Total *	Expected	Hits *	Raw *p* *	Holm *p* *	FDR *p* *	Impact *
1	Biosynthesis of unsaturated fatty acids	22	0.21	2	1.72 × 10^−2^	1.00	1.00	0.00
2	Linoleic acid metabolism	4	0.04	1	3.73 × 10^−2^	1.00	1.00	0.00
3	Sesquiterpenoid and triterpenoid biosynthesis	24	0.23	1	2.05 × 10^−1^	1.00	1.00	0.20374
4	alpha-Linolenic acid metabolism	28	0.26	1	2.35 × 10^−1^	1.00	1.00	0.00
5	Diterpenoid biosynthesis	28	0.26	1	2.35 × 10^−1^	1.00	1.00	0.07625
6	Glycerophospholipid metabolism	37	0.35	1	2.99 × 10^−1^	1.00	1.00	0.07614
7	Ubiquinone and other terpenoid-quinone biosynthesis	38	0.36	1	3.06 × 10^−1^	1.00	1.00	0.02227
8	Steroid biosynthesis	45	0.42	1	3.52 × 10^−1^	1.00	1.00	0.02644
9	Fatty acid biosynthesis	56	0.56	1	4.18 × 10^−1^	1.00	1.00	0.00

* Total is the total number of compounds in the pathway; the Hits is the actually matched number from the user uploaded data; the Raw *p* is the original *p* value calculated from the enrichment analysis; the Holm *p* is the *p* value adjusted by Holm-Bonferroni method; the FDR *p* is the *p* value adjusted using False Discovery Rate; the Impact is the pathway impact value calculated from pathway topology analysis.

**Table 4:** Fold change analysis results in the hexane and CHCl_3_/MeOH extracts.

Metabolites	Molecular Formula	Accurate Mass	Up-Regulation [Hexane Extract]	Up-Regulation [CHCl_3_/MeOH Extract]	Biological Activity/ References
**HeLa, A549 and/or related cell lines**					
*α*-Tocopherolquinone	C_29_H_50_O_3_	446.3760	Yes	-	[23]
Alpinumisoflavone dimethyl ether	C_22_H_20_O_5_	364.1311	Yes	-	H2108 (IC_50_ = 33.5 µM); H1299 (IC_50_ = 38.8 µM) [36]
7,9,13,17-tetramethyl-7*S*,14*S*-dihydroxy-2*E*,4*E*,8*E*,10*E*,12*E*,16-octadecahexaenoic acid	C_22_H_32_O_4_	360.2301	Yes	-	
Heneicosan-2-one	C_21_H_42_O	310.3236	Yes	-	
Gancaonin R	C_24_H_30_O_4_	382.2144	Yes	-	
Calycanthidine	C_23_H_28_N_4_	360.2314	Yes	-	
2-Heptadecylfuran	C_21_H_38_O	306.2923	Yes	-	
Phytol	C_20_H_40_O	296.3079	Yes	-	Hela (IC_50_ = 15.51 ± 0.76 µM); A549 (IC_50_ = 56.98 ± 2.68 µM) [45]
Yucalexin B16	C_20_H_28_O_2_	300.2089	Yes	-	
*δ*-Tocopherol	C_27_H_46_O_2_	402.3498	Yes	-	[46]
(3*E*)-4-(2,3-dihydroxy-2,5,5,8*α*-tetramethyl-decahydronaphthalen-1-yl)but-3-en-2-one	C_18_H_30_O_3_	294.2195	Yes	-	
3*β*,18*β*-3-Methoxy-11-oxo-12-oleanen-30-oic acid	C_31_H_48_O_5_	484.7104	Yes	-	
*N*-(3-hydroxy-dodecanoyl)-homoserine lactone	C_16_H_29_NO_4_	299.2097	Yes	-	[37]
10-methoxyheptadec-1-en-4,6-diyne-3,9-diol	C_18_H_28_O_3_	292.2038	Yes	-	
Lucidone A	C_24_H_34_O_5_	402.2406	Yes	-	
Stylisterol A	C_28_H_46_O_3_	430.3447	Yes	-	HeLa (IC_50_ = 14.1 µM) [38]
Cucurbitacin E	C_32_H_44_O_8_	556.3036	Yes	-	[26]
gibberellin A12 aldehyde	C_20_H_28_O_3_	316.2038	Yes	-	[39]
*α*-Amyrin acetate	C_32_H_52_O_2_	468.3967	Yes	-	[27]
*Ent*-9-L1-phytoP	C_18_H_28_O_4_	308.1988	Yes	-	[47]
*γ*-Linolenic Acid	C_18_H_30_O_2_	278.2246	Yes	-	[31]
(−)-Folicanthine	C_24_H_30_N_4_	374.2470	Yes	-	A549 (IC_50_ = 7.76 µM) [48]
Amabiline	C_15_H_25_NO_4_	283.1784	Yes	-	
Grandifloric acid	C_20_H_30_O_3_	318.2194	Yes	-	[34]
Yucalexin B5	C_20_H_26_O_3_	314.1881	Yes	-	
7′,8′-Dihydro-8′-hydroxycitraniaxanthin	C_33_H_44_O_3_	488.3290	Yes	-	
5,6-Epoxy-5,6-dihydro-10′-apo-*β*,*γ*-carotene-3,10′-diol	C_27_H_38_O_3_	410.2820	Yes	-	
*N*-tetradecanoyl glutamine	C_19_H_36_N_2_O_4_	356.2675	Yes	-	
2-Stearyl citric acid	C_24_H_44_O_7_	444.3087	Yes	-	
17-phenyl heptadecanoic acid	C_23_H_38_O_2_	346.2872	Yes	-	
**PC3, MCF7 and/or related cell lines**					
4′-*O*-Geranylisoliquiritigenin	C_25_H_28_O_4_	392.1988	-	Yes	MDB-MB-231 (IC_50_ = 125.5 µM) [49]
1-Monoacylglycerol	C_20_H_34_NO_4_	352.2619	-	Yes	
Sanguisorbin B	C_35_H_56_O_7_	588.4026	-	Yes	
Oxyacanthine	C_37_H_40_N_2_O_6_	608.2886	-	Yes	[32]
3-Methyl-5-pentyl-2-furannonanoic acid	C_19_H_32_O_3_	308.2351	-	Yes	
2-Monopalmitoylglycerol	C_19_H_38_O_4_	330.2771	-	Yes	
15-hydroxy-5,9-dimethyl-14-methylidenetetracyclo[11.2.1.01,10.04,9]hexadecane-5-carboxylic acid	C_20_H_30_O_3_	318.2194	-	Yes	
4-hydroxy-8-*cis*-sphingenine	C_18_H_37_NO_3_	315.2773	-	Yes	
*N*-(5-aminopentyl)-*N*’-(5-{[4-({5-[butylidene(oxido)-lambda(5)-azanyl]pentyl}amino)-4-oxobutanoyl](hydroxy)amino}pentyl)-*N*-hydroxybutanediamide	C_27_H_52_N_6_O_7_	572.3897	-	Yes	
1,2-di-(9*Z*-pentadecenoyl)-*sn*-glycerol	C_33_H_60_O_5_	536.4441	-	Yes	
4,2′,4′-Trihydroxy-3′,5′-diprenylchalcone	C_25_H_28_O_4_	392.1988	-	Yes	[50]
5,7,4′-Trimethoxyflavan	C_18_H_20_O_4_	300.1362	-	Yes	
all-*trans*-Heptaprenyl diphosphate	C_35_H_60_O_7_P_2_	654.3814	-	Yes	
Heliotrine	C_16_H_27_NO_5_	313.1889	-	Yes	
(22*E*,24*R*)-Stigmasta-4,22-diene-3,6-dione	C_29_H_44_O_2_	424.3341	-	Yes	
Methyl 9*R*-hydroxy-10*E*,12*E*-octadecadienoate	C_19_H_34_O_3_	310.2508	-	Yes	[51]
1,2-di-(9*Z*,12*Z*-octadecadienoyl)-*sn*-glycerol	C_39_H_68_O_5_	616.5067	-	Yes	
*N*-(1,3-dihydroxypropan-2-yl)hexadecanamide	C_21_H_30_O_3_	330.2195	-	Yes	
2-Hydroxy-6-tridecylbenzoic acid	C_20_H_32_O_3_	320.2351	-	Yes	MDA-MB-231 (IC_50_ = 117.25 µM); 4T-1 (IC_50_ = 102.39 µM) [42]
*β*-isorenieratane	C_40_H_72_	552.5634	-	Yes	
Matricin	C_17_H_22_O_5_	306.1467	-	Yes	[35]
9,10,13-trihydroxy-octadecanoic acid	C_18_H_36_O_5_	332.2563	-	Yes	
Austroinulin	C_20_H_34_O_3_	322.2508	-	Yes	
Butyl 3-*O*-*β*-D-glucopyranosyl-butanoate	C_14_H_26_O_8_	322.1628	-	Yes	
Presqualene diphosphate	C_30_H_52_O_7_P_2_	586.3188	-	Yes	
2-*O*-(*β*-D-galactopyranosyl-(1->6)-*β*-D-galactopyranosyl) 2*S*,3*R*-dihydroxynonanoic acid	C_21_H_38_O_14_	514.2262	-	Yes	
Catechin 3-*O*-rutinoside	C_27_H_34_O_15_	598.1898	-	Yes	[52]
Stearic acid	C_18_H_36_O_2_	284.2715	-	Yes	[33]
1-*O*-(2*R*-methoxy-4*Z*-heneicosenyl)-*sn*-glycerol	C_25_H_50_O_4_	414.3709	-	Yes	
Ammothamnidin	C_25_H_28_O_5_	408.1937	-	Yes	
6-Deoxohomodolichosterone	C_29_H_50_O_4_	462.3709	-	Yes	
1-hexadecyl-glycero-3-phospho-(1′-*sn*-glycerol)	C_22_H_47_O_8_P	470.3009	-	Yes	
28-Glucopyranosyl-3-methyloleanolic acid	C_37_H_60_O_8_	632.4285	-	Yes	
Stigmastane-3,6-dione	C_29_H_48_O_2_	428.3654	-	Yes	
N-(dodecanoyl)-sphing-4-enine	C_30_H_59_NO_3_	481.4503	-	Yes	
Phyllospadine	C_21_H_21_NO_6_	383.1369	-	Yes	
Tricosylic acid	C_23_H_46_O_2_	354.3498	-	Yes	
1-Docosahexaenoyl-2-erucoyl-*sn*-glycero-3-phosphocholine	C_52_H_90_NO_8_P	887.6404	-	Yes	

## Data Availability

The data presented in this study are available on request from the corresponding authors.

## Supplementary Materials

The following are available online at https://www.mdpi.com/article/10.3390/plants10071389/s1, Table S1: UHPLC-QTOF-MS/MS results for Hexane extract, Table S2: UHPLC-QTOF-MS/MS results for chloroform/methanol extract, Table S3: UHPLC-QTOF-MS/MS results for aqueous extract, Table S4: Common compounds in the Hexane and CHCl3-MeOH extracts.
